# Identification of adenoid subtype characterized with immune-escaped phenotype in lung squamous carcinoma based on transcriptomics

**DOI:** 10.1186/s40164-022-00327-5

**Published:** 2022-10-12

**Authors:** Jie Mei, Yun Cai, Ofek Mussafi, Mingfeng Zheng, Yongrui Xu, Ruo Chen, Guanyu Jiang, Wenjun Mao, Wei Xia, Yuan Wan

**Affiliations:** 1 Department of Thoracic Surgery, The Affiliated Wuxi People’s Hospital of Nanjing Medical University, No. 299 Qingyang Road, Wuxi, 214023 China; 2 Department of Oncology, The Affiliated Wuxi People’s Hospital of Nanjing Medical University, Wuxi, 214023 China; 3 Wuxi Clinical Medical College, Nanjing Medical University, Wuxi, 214023 China; 4 The Pq Laboratory of BiomeDx/Rx, Department of Biomedical Engineering, Binghamton University, 65 Murray Hill Road, Biotechnology Building BI2625, Binghamton, NY 13850 USA; 5 Department of Intensive Care Unit, The Affiliated Wuxi People’s Hospital of Nanjing Medical University, No. 299 Qingyang Road, Wuxi, 214023 China

**Keywords:** NSCLC, Subtype, Immuno-escaped, Biomarker

## Abstract

Non-small cell lung cancer (NSCLC) is a heterogeneous disease, and its demarcation contributes to various therapeutic outcomes. However, a small subset of tumors shows different molecular features that are in contradiction with pathological classification. Unsupervised clustering was performed to subtype NSCLC using the transcriptome data from the TCGA database. Next, immune microenvironment features of lung adenocarcinoma (LUAD), lung squamous carcinoma (LUSC), and lung adenoid squamous carcinoma (LASC) were characterized. In addition, diagnostic biomarkers to demarcate LASC among LUSC were screened using weighted gene co-expression network analysis (WGCNA) and validated by the in-house cohort. LASC was identified as a novel subtype with adenoid transcriptomic features in LUSC, which exhibited the most immuno-escaped phenotype among all NSCLC subtypes. In addition, FOLR1 was identified as a biomarker for LASC discrimination using the WGCNA analysis, and its diagnostic value was validated by the in-house cohort. Moreover, FOLR1 was related to immuno-escaped tumors in LUSC but not in LUAD. Overall, we proposed a novel typing strategy in NSCLC and identified FOLR1 as a biomarker for LASC discrimination.

**Supplementary Information:**

The online version contains supplementary material available at 10.1186/s40164-022-00327-5.

**To the editor,**

Lung cancer is the leading cause of cancer-related death worldwide. Its histological and biological heterogeneity contributes to various therapeutic outcomes [1]. Lung cancer can be mainly classified as small cell lung cancer (SCLC) and non-SCLC (NSCLC). NSCLC, accounting for about 85% of the lung cancer cases, dominatingly consists of lung adenocarcinoma (LUAD) and lung squamous carcinoma (LUSC) [2]. Although LUAD and LUSC are the largest NSCLC subgroups, they appear to be disparate diseases with distinct molecular, pathological, and clinical features [3]. For example, LUAD seems to be more immune-escaped compared with LUSC. Tumor-infiltrating lymphocytes (TILs) and PD-L1 expression are shown to be higher in LUAD. However, TILs and PD-L1 expression showed inter- and intra-tumor heterogeneity in both LUAD and LUSC [4]. However, due to the heterogeneity, a small subset of tumors shows different molecular features that are in contradiction with pathological classification.

Considering the limited treatment options for LUSC with LUAD [5], we sought to identify a subtype in LUSC with the genomic signatures similar to LUAD. Firstly, we performed unsupervised clustering of LUAD and LUSC patients and identified four clusters (Additional file 2: Fig. S1A, B). Notably, we found that several LUSC patients mingled in clusters enriched with LUAD patients (Additional file 2: Fig. S1C), and we defined these LUSC as lung adenoid squamous carcinoma (LASC) (Fig. 1A). Notably, KRT7, KRT18, and NAPSA, selectively expressed in LUAD, were highly expressed in LASC, while KRT5, TP63, and DSG3, the biomarkers for LUSC, were lowly expressed in LASC (Fig. 1B, C). In addition, critical targetable mutations were also higher in LASC compared with LUSC (Fig. 1D). Previous study showed that oncogenic mutations in EGFR, KRAS, BRAF, HER2, and ALK were extremely rare or absent in patients with pure LUSC, whereas LUSC with minor glandular component (LUSC-mGC) had a relatively high frequency of EGFR, ALK, or KRAS mutations [6], similar genomic alternations to the third subtype of LASC found in our study. However, whether LASC belongs to LUSC-mGC is need to be further explored. Moreover, the prognosis of LASC was worse than LUSC in terms of overall survival (OS), progression-free survival (PFS), and disease-special survival (DSS) (Fig. 1E). Given difference in TILs between LUAD and LUSC [4], we next characterized the immune features in three NSCLC subtypes. LASC exhibited the highest immune score, stromal score, and ESTIMATE score, while the lowest tumor purity (Additional file 2: Fig. S2A). In addition, most immune-modulators and most TILs were the highest in LASC, followed by LUAD (Additional file 2: Fig. S2B, C). Furthermore, the most immune checkpoints were the highest in LASC (Additional file 2: Fig. S2D).

**Fig. 1:**
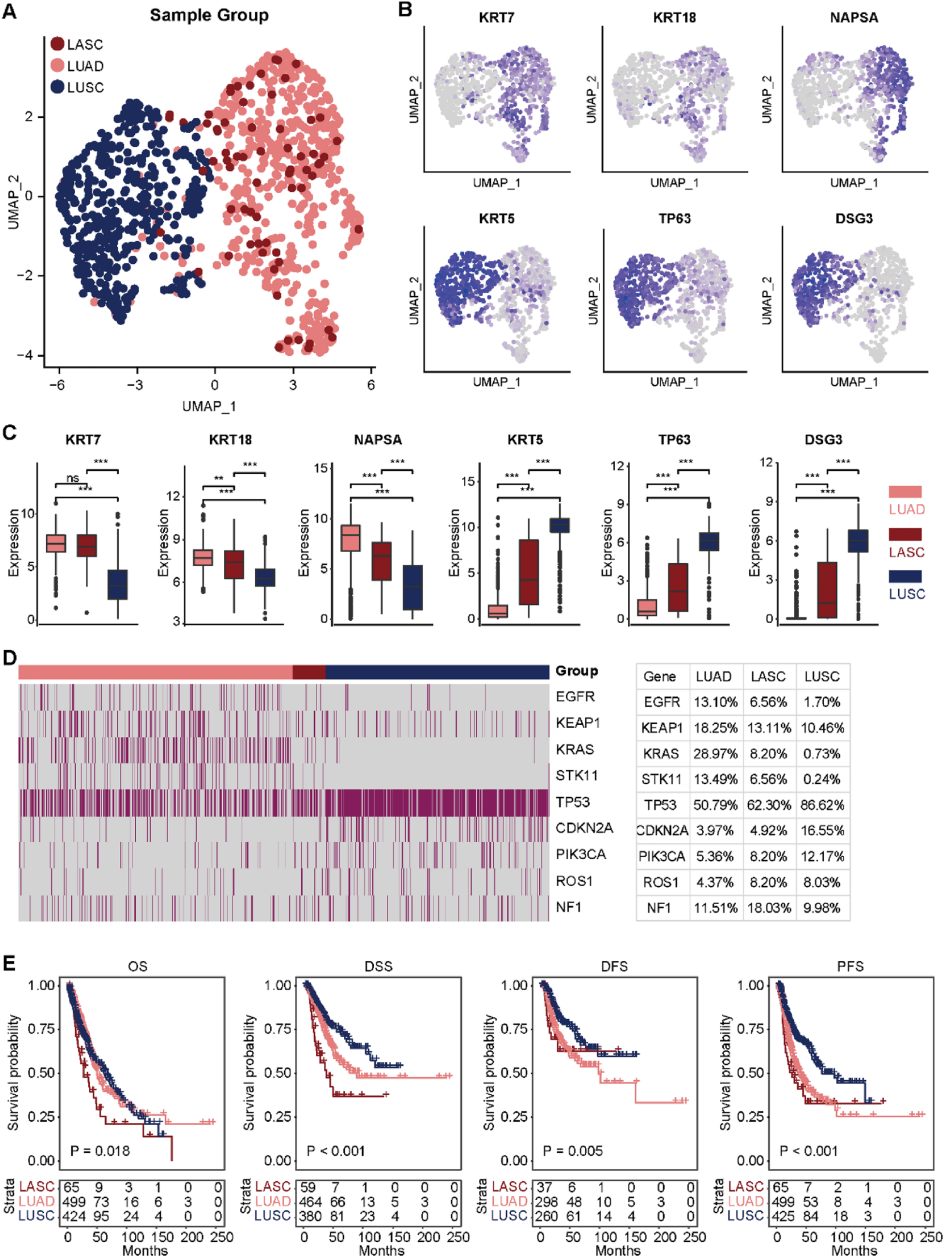
Identification of LASC as a novel subtype in LUSC. **A** Unsupervised clustering of LUAD, LUSC, and LASC samples. **B**, **C** Expression levels of KRT7, KRT18, NAPSA, KRT5, TP63, and DSG3 in LUAD (n = 512), LUSC (n = 430), and LASC (n = 66) samples. Significance was calculated with One-way ANOVA with Tukey’s multiple comparisons test. *ns* no statistical difference, **P < 0.01, ***P < 0.001. **D** Mutant profiles of EGFR, KEAP1, KRAS, STK11, TP53, CDKN2A, PIK3CA, ROS1, and NF1 in LUAD, LUSC, and LASC samples. **E** Prognostic analysis of patients in LUAD, LUSC, and LASC subtypes. Significance was calculated with log-rank test

We next explored discriminating biomarkers for LASC using the WGCNA algorithm (Additional file 2: Fig. S3A–D). We visualized the gene network with a heatmap and meta-modules ((Additional file 2: Fig. S4A, B), and two modules were extracted (Additional file 2: Fig. S4C). The genes in the turquoise and blue modules were mainly associated with tumor immunity-related with processes and surfactant homeostasis, respectively (Additional file 2: Fig. S4D, E). Given genes in the turquoise module were TIL markers, we utilized the genes in the blue module as biomarkers for LASC. The score of these genes was highly expressed in LASC compared with LUSC and exhibited high diagnostic values (Fig. 2A, B). In addition, FOLR1 exhibited the highest value among these genes (Fig. 2C). Moreover, the results from the validated cohort showed that FOLR1 was upregulated in LUAD compared with LUSC (Fig. 2D, E), which could be a novel biomarker in the discrimination between LUAD and LUSC. In addition, high FOLR1 was associated with poor prognosis in LUSC (Fig. 2F).

**Fig. 2:**
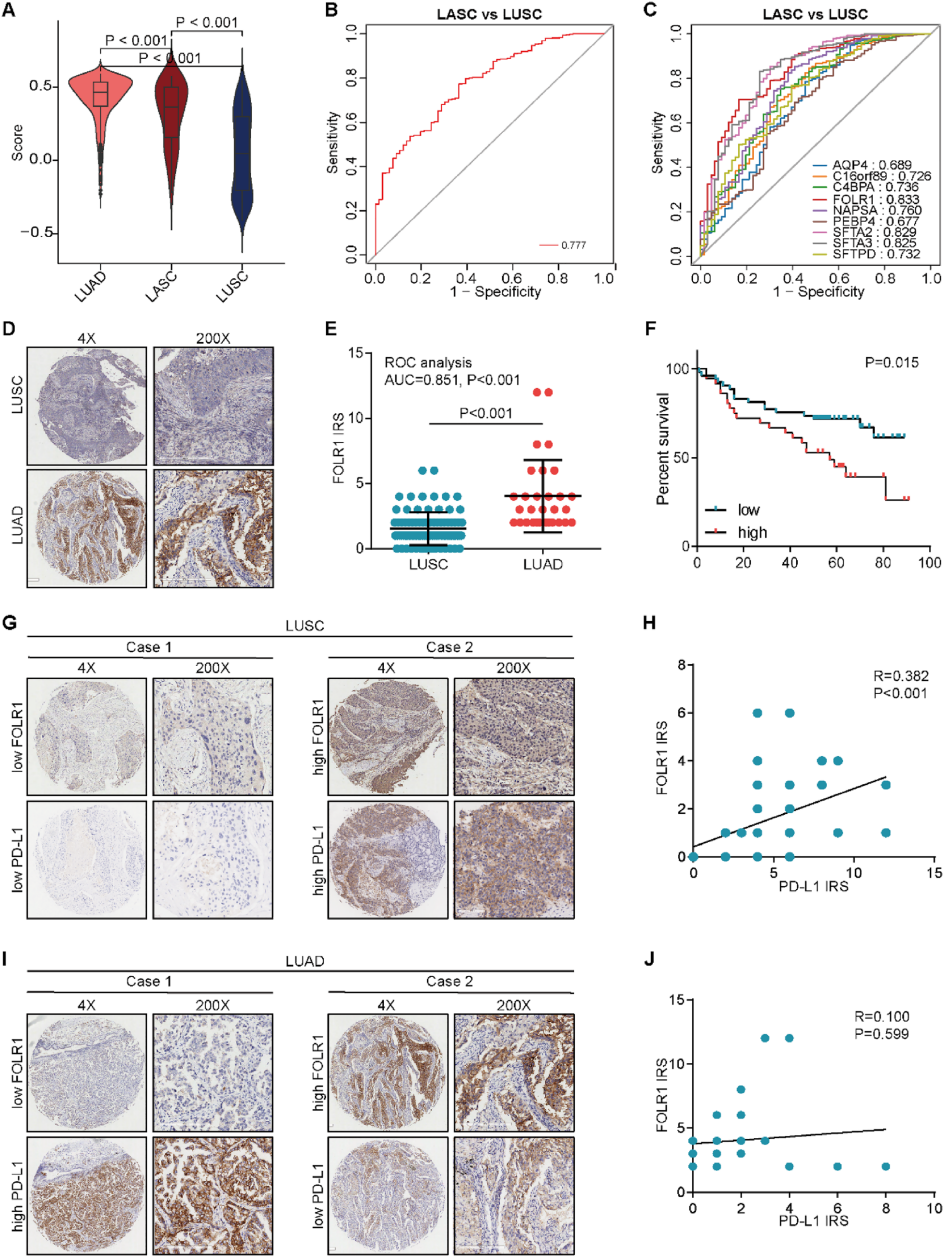
FOLR1 is a biomarker for LASC discrimination and correlated immune feature in LUSC. **A** Levels of the score of genes in the blue calculated by the ssGSEA method in LUAD (n = 512), LUSC (n = 430), and LASC (n = 66) subtypes. Significance was calculated with One-way ANOVA with Tukey’s multiple comparisons test. **B** Diagnostic value of the score of genes in the blue for the discrimination LASC in LUSC. **C** Diagnostic value of the single gene in the blue for the discrimination LASC in LUSC. **D**, **E** Representative images revealing FOLR1 expression in LUAD (n = 30) and LUSC (n = 90) subtypes and semi-quantitative analysis. Significance was calculated with Student’s t-test. **F** Prognostic value of FOLR1 expression in LUSC. Fifty-three patients with low FOLR1 expression, and 47 patients with high FOLR1 expression. Significance was calculated with log-rank test. **G** Representative images revealing low and high FOLR1 and PD-L1 expression in LUSC. **H** Correlation between FOLR1 and PD-L1 expression in LUSC. Significance was calculated with Pearson test. **I** Representative images revealing low and high FOLR1 and PD-L1 expression in LUAD. **J** Correlation between FOLR1 and PD-L1 expression in LUAD. Significance was calculated with Pearson test

Subsequently, we investigated the correlation of FOLR1 with immune features. In LUSC, immune score, stromal score, and ESTIMATE score were higher, while tumor purity was lower in the high-FOLR1 group (Additional file 2: Fig. S5A). In addition, most immune-modulators and TILs were enriched in the high-FOLR1 group (Additional file 2: Fig. S5B, C). Moreover, FOLR1 was positively correlated with most immune checkpoints (Additional file 2: Fig. S5D). However, these correlations could not be observed in LUAD (Additional file 2: Fig. S6A–D). The results from the validated cohort showed that FOLR1 was positively correlated with PD-L1 in LUSC but not in LUAD (Fig. 2G**–**J). We also assessed the expression of FOLR1 in different cell types in NSCLC and the results showed that FOLR1 was highly expressed in tumor cells (Additional file 2: Fig. S7A, B). FOLR1 is often overexpressed in multiple cancers, which is often associated with tumor progression and poor patient prognosis [7]. In lung cancer, FOLR1 is mainly expressed in LUAD and more highly expressed in metastatic lymph node, and nanoparticle targeted FOLR1 enhanced photodynamic therapy [8]. In addition, high FOLR1 expression correlates with adenocarcinoma histology and EGFR mutation in lung cancer [9]. Furthermore, trans-differentiation of LUAD to LUSC depending on the signaling of Lkb1 [10], Whether FOLR1 mediates trans-differentiation between LUAD and LUSC and its association with Lkb1 signaling might be an interesting topic.

Immune feature-based risk stratification is critical for prognostic and therapeutic assessment in both lung cancer and other cancer types [11–14]. To sum up, we proposed a novel typing strategy in NSCLC and indicated LASC could be a dominant subtype benefiting from immunotherapy. We also identified FOLR1 as a biomarker for LASC discrimination. However, due to limited clinical features and genetic alterations provided by Outdo Biotech, we fail to compare the associations between FOLR1 expression and clinical features in detail, which should be further explored. Overall, the importance of FOLR1 detection should be emphasized in LUSC for the identification of the novel subtype.

## Supplementary Information

**Additional file 1.** Additional methods.

**Additional file 2: Figure S1.** Demarcation of patients using a SNN modularity optimization-based clustering algorithm. **A** Unsupervised clustering of NSCLC samples with four clusters. **B** Unsupervised clustering of LUAD and LUSC samples. **C** Distribution of LUAD and LUSC samples in four different clusters. **Figure S2.** Associations between NSCLC subtypes and TME features. **A** Levels of stromal score, immune score, ESTIMATE score, and tumor purity in LUAD (n = 512), LUSC (n = 430), and LASC (n = 66) subtypes. Significance was calculated with One-way ANOVA with Tukey’s multiple comparisons test. ***P < 0.001. **B** Expression levels of 122 immunomodulators in LUAD, LUSC, and LASC subtypes. **C** The levels of TILs calculated using five algorithms in LUAD, LUSC, and LASC subtypes. **D** Expression levels of immune checkpoints in LUAD, LUSC, and LASC subtypes. **Figure S3.** Determination of soft-thresholding power in WGCNA. **A** Analysis of the scale-free fitting indices for various soft-thresholding powers (β). **B** Mean connectivity analysis of various soft-thresholding powers. **C** Histogram of the connection distribution when β = 14. **D** Checking the scale-free topology when β = 14. According to Figure S3C-D, k and p(k) are negatively correlated (correlation coefficient 0.78), indicating that a gene scale-free network can be resumed. **Figure S4.** Identification of FOLR1 as a biomarker for LASC discrimination. **A** Visualization of the gene network with a heatmap. **B** Clustering dendrograms of genes based on dissimilarity topological overlap and module colors. **C** Heatmap of the correlation between module eigengenes and subtypes of NSCLC. **D** BP enrichment analysis of genes in the turquoise module. **E** BP enrichment analysis of genes in the blue module. **Figure S5.** FOLR1 identifies the inflamed TME in LUSC. **A** Levels of stromal score, immune score, ESTIMATE score, and tumor purity in the high- (n = 182) and low-FOLR1 (n = 314) groups. Significance was calculated with Student’s t-test. ***P < 0.001. **B** Expression levels of 122 immunomodulators in the high- and low-FOLR1 groups. **C** The levels of TILs calculated using five algorithms in the high- and low-FOLR1 groups. **D** Correlations between FOLR1 and common inhibitory immune checkpoints. ***P-value < 0.001. **Figure S6.** FOLR1 can’t identify the inflamed TME in LUAD. **A** Levels of stromal score, immune score, ESTIMATE score, and tumor purity in the high- (n = 188) and low-FOLR1 (n = 324) groups. Significance was calculated with Student’s t-test. ns: no statistical difference, *P < 0.05. **B** Expression levels of 122 immunomodulators in the high- and low-FOLR1 groups. (C) The levels of TILs calculated using five algorithms in the high- and low-FOLR1 groups. **D** Correlations between FOLR1 and common inhibitory immune checkpoints.  *P-value < 0.05; ns: P > 0.05. **Figure S7.** FOLR1 is highly expressed in tumor cells in NSCLC. **A** Single-cell expression profile of FOLR1 in the GSE117570 dataset. **B** Single-cell expression profile of FOLR1 in the GSE131907 dataset.

## Data Availability

All data supported the results in this study are showed in this published article and its Additional files 1 and 2. In addition, original data for bioinformatics analysis could be downloaded from corresponding platforms.

## Abbreviations

LASC: Lung adenoid squamous carcinoma.
LUAD: Lung adenocarcinoma
LUSC: Lung squamous carcinoma
LUSC-mGC: LUSC with minor glandular component
NSCLC: Non-small cell lung cancer
SCLC: Small cell lung cancer
TIL: Tumor-infiltrating lymphocyte
