# Supplementary material for: Identification of adenoid subtype characterized with immune-escaped phenotype in lung squamous carcinoma based on transcriptomics
Source: Exp Hematol Oncol. 2022 Oct 12;11:70. doi: 10.1186/s40164-022-00327-5 (PMC9555124; doi:10.1186/s40164-022-00327-5)
Supplement: Supplementary file 1 — Additional file 1. Additional methods. [file 40164_2022_327_MOESM1_ESM.docx]

**Supplementary Methods**

**Public data acquisition and bioinformatics analysis**

The RNA-sequencing (RNA-seq) data of NSCLC (IlluminaHiSeq, log_2_(x+1) transformed RSEM normalized count) and corresponding clinical information in The Cancer Genome Atlas (TCGA) database was downloaded from the UCSC Xena portal (http://xenabrowser.net/datapages/). The public data was utilized to define the subtype of NSCLC, investigate the immunological features, and identify potential diagnostic biomarkers.

**Subtyping NSCLC**

Unsupervised clustering was performed to subtype NSCLC at transcriptome levels. Firstly, the top 2,000 genes with the highest variance were used for principal component analysis (PCA) to reduce dimensionality. Then, the dimensionality of the expression profile matrix was further reduced to two-dimensional space based on the first 20 principal components (PCs) and visualized by uniform manifold approximation and projection (UMAP). The clusters were identified based on a shared nearest neighbor (SNN) modularity optimization-based clustering algorithm with a resolution of 0.3, and all patients were divided into four clusters.

**Description of immunological features**

The immunological characteristics of the tumor microenvironment (TME) in NSCLC contained ESTIMATE scores, immunomodulators, levels of tumor-infiltrating lymphocyte (TILs), and the expressions of immune checkpoints. Firstly, the ESTIMATE algorithm was conducted to estimate Tumor Purity, ESTIMATE Score, Immune Score, and Stromal Score [1]. In addition, the expressions of 122 immune modulators, including MHC, receptors, chemokines, and immuno-stimulating factors, were also regarded as the immunological characteristics of TME [2]. To avoid the calculation error caused by various algorithms, we used four independent algorithms: TIMER [3] EPIC [4], MCP-counter [5], and TISIDB [6]. Moreover, the expression levels of immune checkpoints were also evaluated.

**Weighted gene co-expression network analysis (WGCNA)**

WGCNA is a systematic biological strategy for evaluating gene association patterns among different samples [7]. To conduct WGCNA, the DEGs between LASC and LUSC with the criterion of FC≥2 and P<0.05 were selected to construct a gene co-expression network using the WGCNA package in R [7]. The idea of a soft threshold is to continually elementize the elements in the Adjacency Matrix through a weight function. Because the choice of the soft threshold, β, is bound to affect the result of module identification and the relative network of the random average of each node, there is a scale-free network in which a few nodes exhibit a significantly higher degree than the general point, which is a more stable choice, so we need to choose a soft threshold, β, is our gene distribution in line with the scale-free network. To create a network with a nearly scale-free topology, we installed the soft threshold power of β = 14 (scale-free R^2^ = 0.90). Adjacency matrices were calculated and transformed into the topological overlap matrix (TOM). The dynamic tree cut algorithm was applied to detect gene modules. Gene significance (GS) was defined as the correlation coefficient between gene expression and module traits. The module eigengene was calculated as a summary profile for each module. Module significance was defined as the correlation coefficient between a module’s eigengene and traits. Module membership (MM) was defined by the correlation coefficient of the module eigengene and gene expression profile. Genes with MM values above 0.90 were considered to be the modules’ representative genes with potential critical functions. In addition, the score of gene expression in a specific module was calculated by single-sample gene set enrichment analysis (ssGSEA).

**Collection of NSCLC specimens**

The LUSC (Cat. HLugS180Su01) and LUAD (Cat. HLugA060PG02) tumor tissue microarrays (TMAs) were purchased from Outdo BioTech (Shanghai, China). A total of 120 paraffin-embedded NSCLC (90 LUSC and 30 LUAD) samples and paired para-tumor samples were contained in the current research. Detailed clinic-pathological and follow-up data were provided by Outdo BioTech. Ethical approval was granted by the Clinical Research Ethics Committee in Outdo Biotech (Shanghai, China).

**Immunohistochemistry (IHC) staining and semi-quantitative assessment**

IHC staining was conducted on the above sections according to the standardized procedures. The sections were then washed with xylene for three 5-min. The sections were rehydrated by successive washes in 100, 90 and 70% graded ethanol. Hydrogen peroxidase was used to block endogenous peroxidase activity for 20 min. The antigen retrieval solution is EDTA. The primary antibodies used were as follows: anti-FOLR1 (1:5000 dilution, Cat. A20726, Abclonal) and anti-PD-L1 (Ready-to-use, Cat. GT2280, GeneTech). Antibody staining was visualized with DAB and hematoxylin counterstain, and stained sections were captured using Aperio Digital Pathology Slide Scanners. The stained sections were independently evaluated by two pathologists. Expression levels of FOLR1 and PD-L1 in tumor cells were semi-quantitatively assessed according to the 12-point criterion by calculating the immunoreactivity score (IRS) [8]. Briefly, the percentage of positively stained cells was scored as 0-4: 0 (< 5%), 1 (6-25%), 2 (26-50%), 3 (51-75%) and 4 (>75%). The staining intensity was scored as 0-3: 0 (negative), 1 (weak), 2 (moderate), and 3 (strong). The IRS equals to the percentages of positive cells multiplied with staining intensity.

**Single cell RNA-seq analysis**

Tumor Immune Single-cell Hub (TISCH, http://tisch.comp-genomics.org/gallery/) is a scRNA-seq database focusing on TME and provides detailed cell-type annotation at the single-cell level [9]. We used TISCH to analyze the cell subpopulation patterns of FOLR1 in NSCLC. Default options were used for all parameters.

**Statistical analysis**

All statistical analyses were conducted using SPSS 26.0 and R 4.0.2. All data are presented as means ± SDs. The difference between the two groups was analyzed by Student’s t-test or Mann Whitney test. Survival analysis was performed by log-rank test. Correlation analysis between two variables was analyzed by the Pearson test. All statistical tests were two-sided, and P-value < 0.05 was considered statistically significant and labeled with *P < 0.05; **P < 0.01; ***P < 0.001.

**Reference**

1. Yoshihara K, Shahmoradgoli M, Martinez E, Vegesna R, Kim H, Torres-Garcia W, et al. Inferring tumour purity and stromal and immune cell admixture from expression data. Nat Commun. 2013;4:2612.

2. Charoentong P, Finotello F, Angelova M, Mayer C, Efremova M, Rieder D, et al. Pan-cancer Immunogenomic Analyses Reveal Genotype-Immunophenotype Relationships and Predictors of Response to Checkpoint Blockade. Cell Rep. 2017;18(1):248-62.

3. Li T, Fu J, Zeng Z, Cohen D, Li J, Chen Q, et al. TIMER2.0 for analysis of tumor-infiltrating immune cells. Nucleic Acids Res. 2020;48(W1):W509-W14.

4. Racle J, de Jonge K, Baumgaertner P, Speiser DE, Gfeller D. Simultaneous enumeration of cancer and immune cell types from bulk tumor gene expression data. Elife. 2017;6.

5. Becht E, Giraldo NA, Lacroix L, Buttard B, Elarouci N, Petitprez F, et al. Estimating the population abundance of tissue-infiltrating immune and stromal cell populations using gene expression. Genome biology. 2016;17(1):218.

6. Ru B, Wong CN, Tong Y, Zhong JY, Zhong SSW, Wu WC, et al. TISIDB: an integrated repository portal for tumor-immune system interactions. Bioinformatics. 2019;35(20):4200-2.

7. Langfelder P, Horvath S. WGCNA: an R package for weighted correlation network analysis. BMC Bioinformatics. 2008;9:559.

8. Mei J, Liu Y, Yu X, Hao L, Ma T, Zhan Q, et al. YWHAZ interacts with DAAM1 to promote cell migration in breast cancer. Cell Death Discov. 2021;7(1):221.

9. Sun D, Wang J, Han Y, Dong X, Ge J, Zheng R, et al. TISCH: a comprehensive web resource enabling interactive single-cell transcriptome visualization of tumor microenvironment. Nucleic acids research. 2021;49(D1):D1420-D30.
