# Supplementary material for: Identification of adenoid subtype characterized with immune-escaped phenotype in lung squamous carcinoma based on transcriptomics
Source: Exp Hematol Oncol. 2022 Oct 12;11:70. doi: 10.1186/s40164-022-00327-5 (PMC9555124; doi:10.1186/s40164-022-00327-5)
Supplement: Supplementary file 2 — Additional file 2: Figure S1. Demarcation of patients using a SNN modularity optimization-based clustering algorithm. A Unsupervised clustering of NSCLC samples with four clusters. B Unsupervised clustering of LUAD and LUSC samples. C Distribution of LUAD and LUSC samples in four different clusters. Figure S2. Associations between NSCLC subtypes and TME features. A Levels of stromal score, immune score, ESTIMATE score, and tumor purity in LUAD (n = 512), LUSC (n = 430), and LASC (n = 66) subtypes. Significance was calculated with One-way ANOVA with Tukey’s multiple comparisons test. ***P < 0.001. B Expression levels of 122 immunomodulators in LUAD, LUSC, and LASC subtypes. C The levels of TILs calculated using five algorithms in LUAD, LUSC, and LASC subtypes. D Expression levels of immune checkpoints in LUAD, LUSC, and LASC subtypes. Figure S3. Determination of soft-thresholding power in WGCNA. A Analysis of the scale-free fitting indices for various soft-thresholding powers (β). B Mean connectivity analysis of various soft-thresholding powers. C Histogram of the connection distribution when β = 14. D Checking the scale-free topology when β = 14. According to Figure S3C-D, k and p(k) are negatively correlated (correlation coefficient 0.78), indicating that a gene scale-free network can be resumed. Figure S4. Identification of FOLR1 as a biomarker for LASC discrimination. A Visualization of the gene network with a heatmap. B Clustering dendrograms of genes based on dissimilarity topological overlap and module colors. C Heatmap of the correlation between module eigengenes and subtypes of NSCLC. D BP enrichment analysis of genes in the turquoise module. E BP enrichment analysis of genes in the blue module. Figure S5. FOLR1 identifies the inflamed TME in LUSC. A Levels of stromal score, immune score, ESTIMATE score, and tumor purity in the high- (n = 182) and low-FOLR1 (n = 314) groups. Significance was calculated with Student’s t-test. ***P < 0.001. B Expression levels [file 40164_2022_327_MOESM2_ESM.docx]

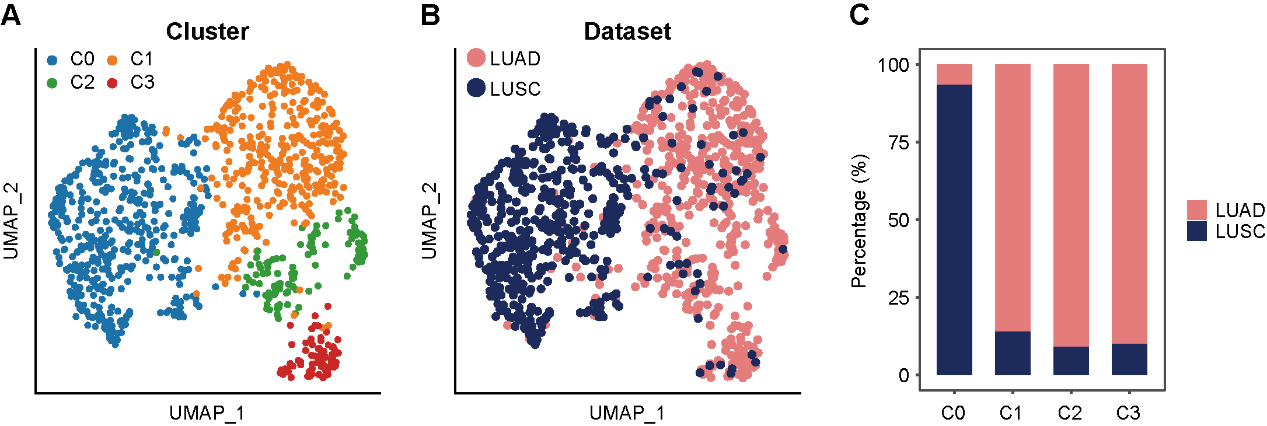


**Figure S1. Demarcation of patients using a SNN modularity optimization-based clustering algorithm.** (A) Unsupervised clustering of NSCLC samples with four clusters. (B) Unsupervised clustering of LUAD and LUSC samples. (C) Distribution of LUAD and LUSC samples in four different clusters.


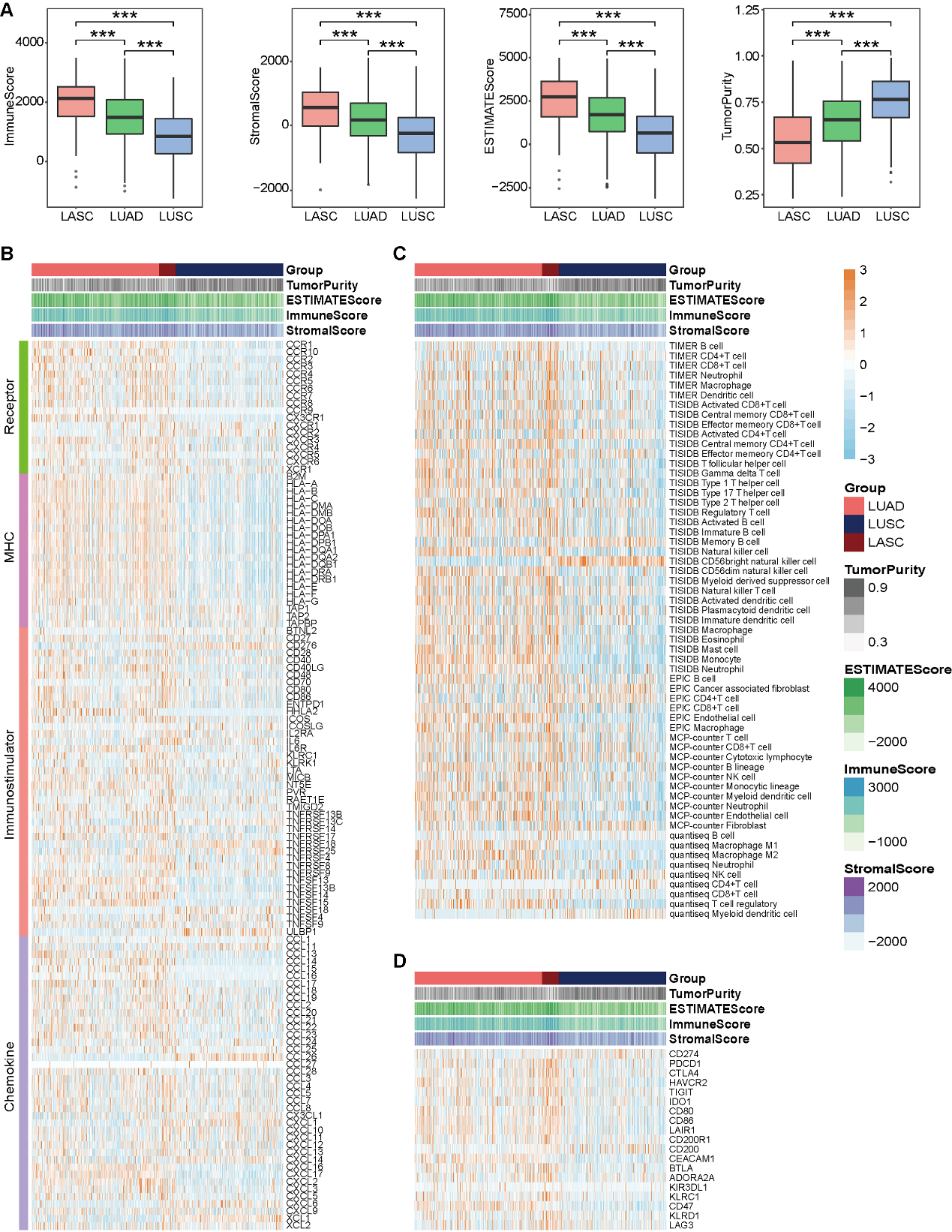


**Figure S2. Associations between NSCLC subtypes and TME features.** (A) Levels of stromal score, immune score, ESTIMATE score, and tumor purity in LUAD (n=512), LUSC (n=430), and LASC (n=66) subtypes. Significance was calculated with One-way ANOVA with Tukey’s multiple comparisons test. ***P<0.001. (B) Expression levels of 122 immunomodulators in LUAD, LUSC, and LASC subtypes. (C) The levels of TILs calculated using five algorithms in LUAD, LUSC, and LASC subtypes. (D) Expression levels of immune checkpoints in LUAD, LUSC, and LASC subtypes.


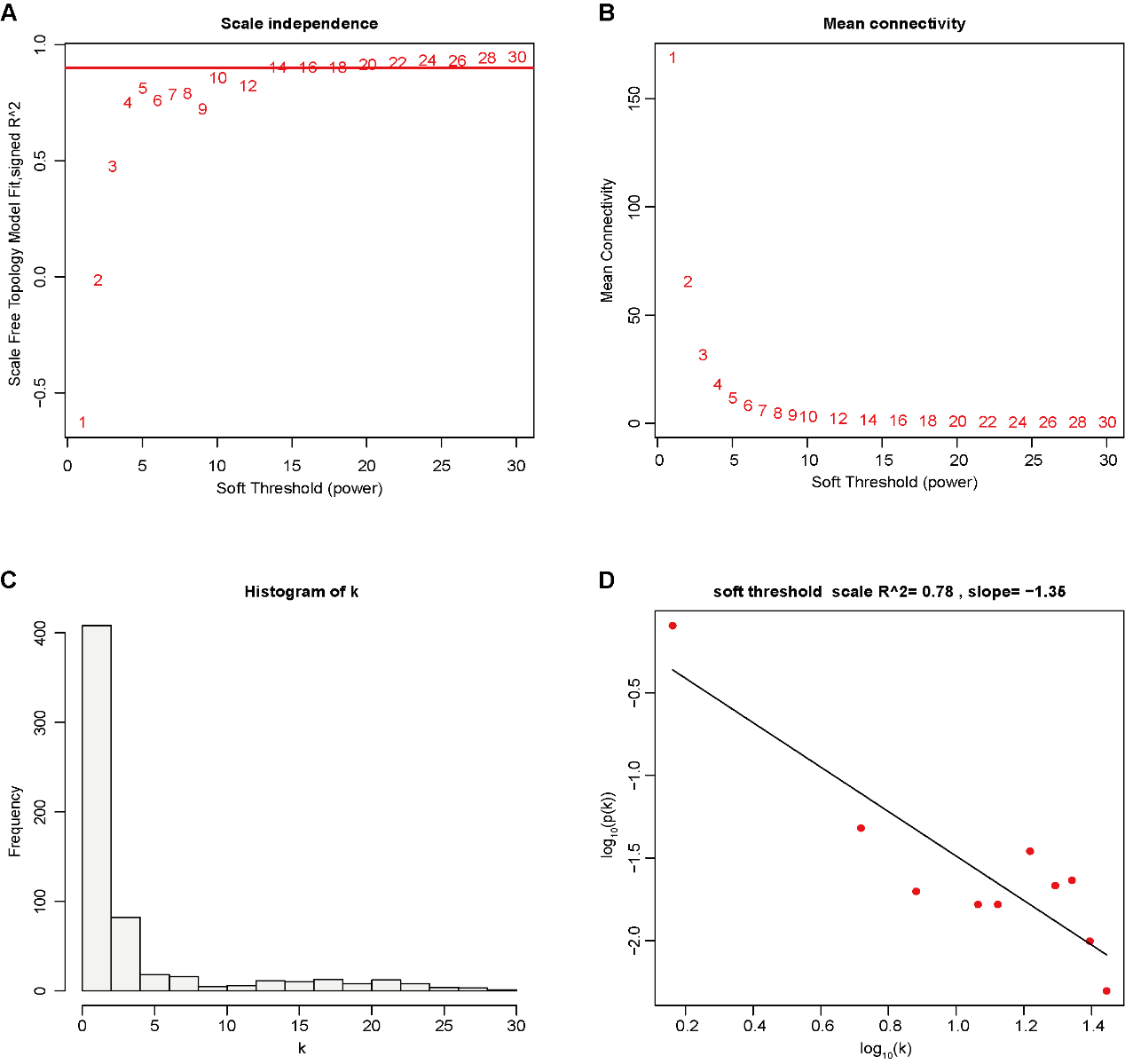


**Figure S3. Determination of soft-thresholding power in WGCNA.** (A) Analysis of the scale-free fitting indices for various soft-thresholding powers (β). (B) Mean connectivity analysis of various soft-thresholding powers. (C) Histogram of the connection distribution when β = 14. (D) Checking the scale-free topology when β = 14. According to Figure S3C-D, k and p(k) are negatively correlated (correlation coefficient 0.78), indicating that a gene scale-free network can be resumed.


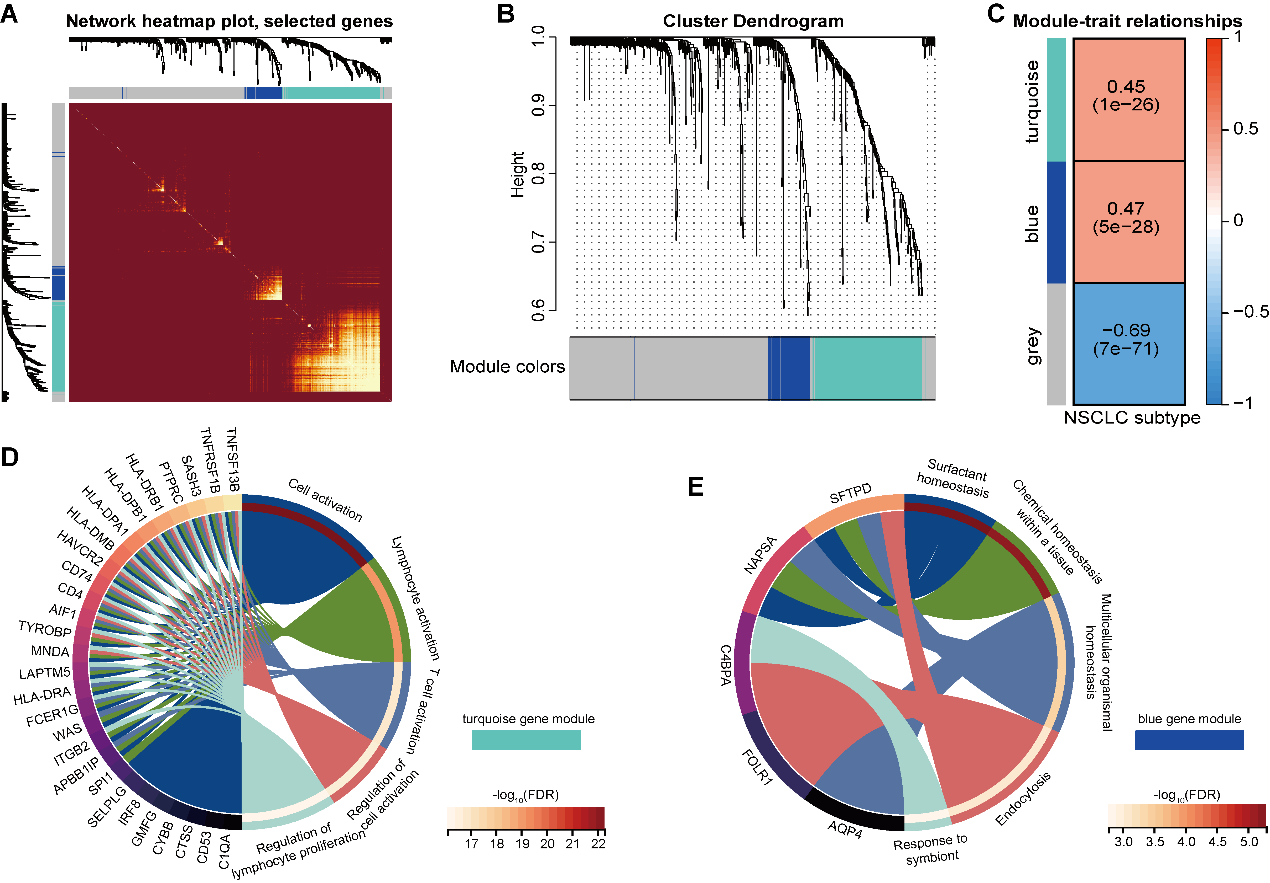


**Figure S4. Identification of FOLR1 as a biomarker for LASC discrimination.** (A) Visualization of the gene network with a heatmap. (B) Clustering dendrograms of genes based on dissimilarity topological overlap and module colors. (C) Heatmap of the correlation between module eigengenes and subtypes of NSCLC. (D) BP enrichment analysis of genes in the turquoise module. (E) BP enrichment analysis of genes in the blue module.


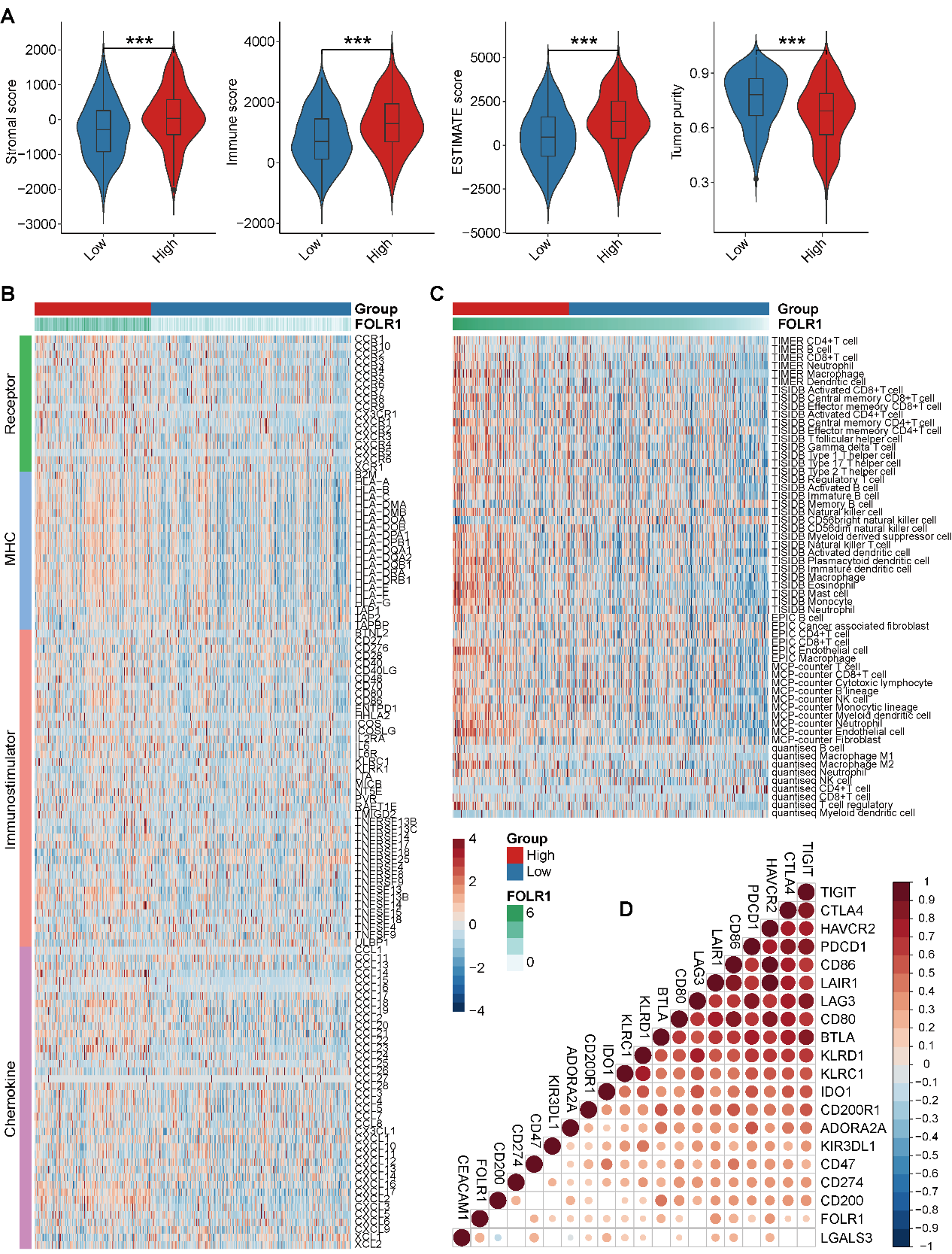


**Figure S5. FOLR1 identifies the inflamed TME in LUSC.** (A) Levels of stromal score, immune score, ESTIMATE score, and tumor purity in the high- (n=182) and low-FOLR1 (n=314) groups. Significance was calculated with Student’s t-test. ***P<0.001. (B) Expression levels of 122 immunomodulators in the high- and low-FOLR1 groups. (C) The levels of TILs calculated using five algorithms in the high- and low-FOLR1 groups. (D) Correlations between FOLR1 and common inhibitory immune checkpoints. Note: ***P-value<0.001.


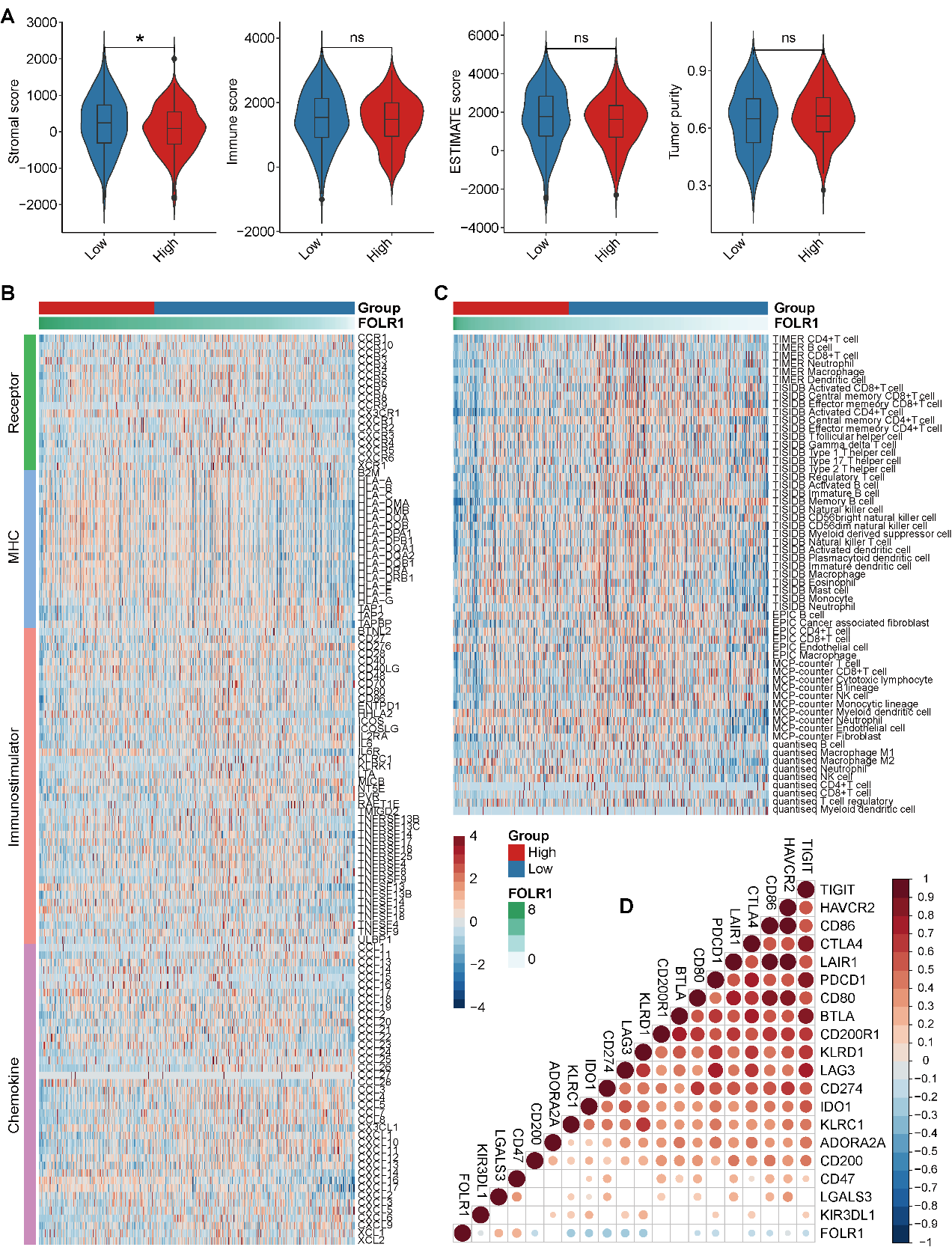


**Figure S6. FOLR1 can’t identify the inflamed TME in LUAD.** (A) Levels of stromal score, immune score, ESTIMATE score, and tumor purity in the high- (n=188) and low-FOLR1 (n=324) groups. Significance was calculated with Student’s t-test. ns: no statistical difference, *P<0.05. (B) Expression levels of 122 immunomodulators in the high- and low-FOLR1 groups. (C) The levels of TILs calculated using five algorithms in the high- and low-FOLR1 groups. (D) Correlations between FOLR1 and common inhibitory immune checkpoints. Note: *P-value<0.05; ns: P>0.05.


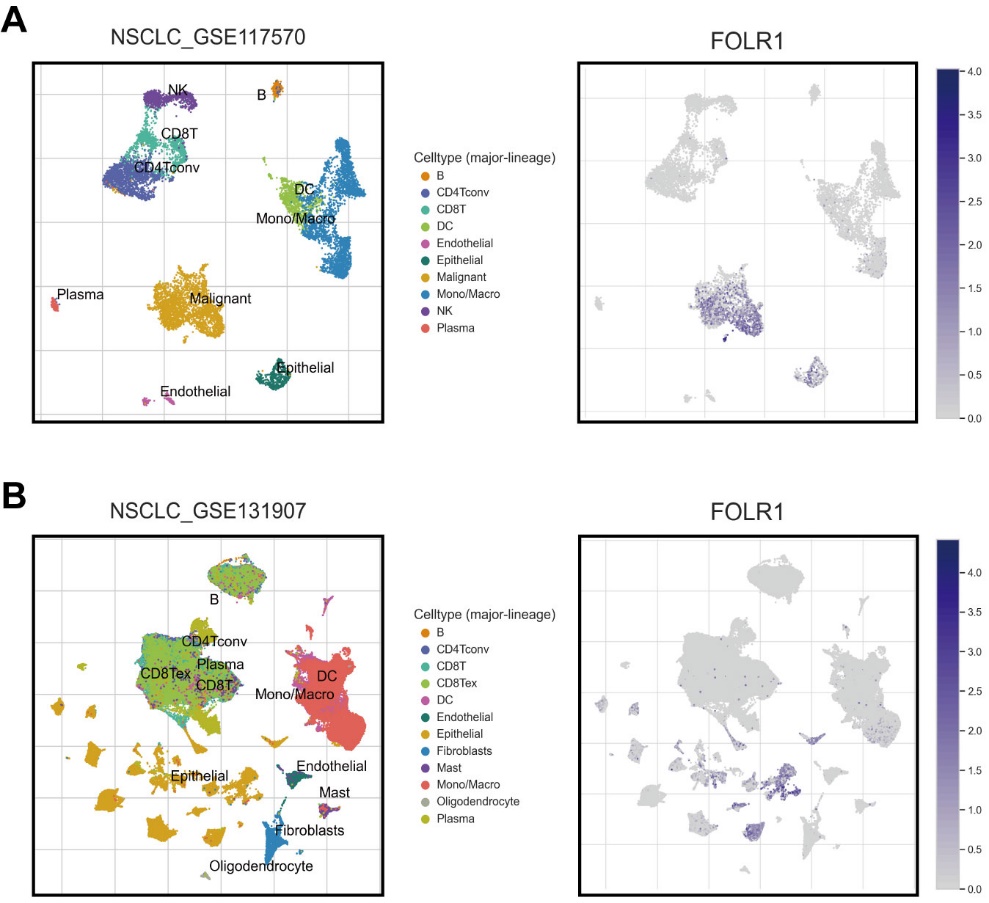


**Figure S7. FOLR1 is highly expressed in tumor cells in NSCLC.** (A) Single-cell expression profile of FOLR1 in the GSE117570 dataset. (B) Single-cell expression profile of FOLR1 in the GSE131907 dataset.
